# Evaluating community pharmacists’ practices in managing insomnia in Saudi Arabia: a cross-sectional study using simulated patient visits

**DOI:** 10.3389/fpubh.2026.1792148

**Published:** 2026-06-16

**Authors:** Mohammad Algarni, Saleh Alghamdi, Bassant Mohamed Barakat, Thamir M Alshammari, Abdullah Alzahrani, Adnan Alzahrani, Hussain Sumayli, Saeed A. Al-Qahtani, Mohammad Jaffar Sadiq Mantargi

**Affiliations:** 1 Department of Clilnical Pharmacy, Faculty of pharmacy, Al-Baha University, Al-Baha, Saudi Arabia; 2 Department of Pharmacy Practice, College of Pharmacy, Jazan University, Jazan, Saudi Arabia; 3 Pharmacy Practice Research Unit, College of Pharmacy, Jazan University, Jazan, Saudi Arabia; 4 Department of Pharmaceutical Sciences, Pharmacy Program, Batterjee Medical College, Jeddah, Saudi Arabia

**Keywords:** community pharmacist, insomnia management, OTC medications, patient counseling, simulated patient, sleep disorder

## Abstract

**Background:**

Insomnia prevails globally and nationally as a serious public health issue with a global prevalence of 48% and is significantly linked to morbidity and productivity loss. Community pharmacists play a vital role in addressing the issue through over-the-counter (OTC) medications and lifestyle advice, yet insomnia management practices remain unexplored in Saudi Arabia. Hence, this study addresses the gap using a simulated patient (SP) methodology.

**Methods:**

A cross-sectional study employing SP was conducted to evaluate community pharmacists’ approach to insomnia management across three regions of Saudi Arabia. A total of 251 visits were identified under the standard scenario. Data collected included pharmacist behavior, consultation duration, and recommendations. Data analysis included descriptive statistics, the chi-square test, Pearson correlation, and binary logistic regression to identify the predictors of pharmacists’ history taking and patient counseling.

**Results:**

A total of 251 simulated visits to pharmacies were completed (urban area 43.4%, suburban area 49.4%, and rural 7.2%), consultation lasted for only 1.5 min (interquartile range [IQR] 1–2 min), most pharmacists inquired about sleep patterns (55.4%) and triggers (51%), recommended OTC medications (e.g., melatonin, 64.5%), and non-pharmacological advice were less frequent (9.6%). Safety information was infrequent (4.9%). Pharmacist professionalism was high (85.7%), but privacy, self-introduction, and referrals were low, with significant regional variation among pharmacies (*p* < 0.001, *χ*^2^). Binary logistic regression identified multiple strong predictors across all domains, including day of visit (odds ratio [OR] = 509.979), interaction duration (OR = 0.338), and key questioning behaviors.

**Discussion:**

Saudi pharmacists opted for safer OTC melatonin for the management of insomnia with brief consultation (IQR = 1–2 min), incomplete history taking, limited safety counseling (<5%), and rare non-pharmacological advice (9.6%). Regional variations and time pressures highlight the need for workflow improvements to enhance patient-centered care. The day of the visit displayed a strong effect (OR = 509.979), whereas longer pharmacist interactions were associated with lower odds of missing key questions (OR = 0.338). Accompanying symptoms (OR = 6567.348) and chronic health issues (OR = 941.751) were identified as strong predictors.

**Conclusion:**

Although pharmacists were accessible, gaps in insomnia management may risk patient safety. Targeted training for pharmacists (continuous professional development [CPD] programs), formulating community guidelines for pharmacists, and systemic changes are important to optimize their role in sleep health.

## 1 Introduction

Insomnia, defined as difficulty initiating or maintaining sleep, is among the most prevalent sleep disorders worldwide. It significantly affects physical health, cognitive function, emotional wellbeing, and overall quality of life. The global burden of insomnia has grown, with prevalence rates ranging from 10 to 30% and being even higher in specific populations such as the older adults, shift workers, and individuals with comorbid psychiatric or medical conditions (1). The World Health Organization (WHO) has recognized sleep disorders, including insomnia, as public health concerns owing to their contribution to non-communicable diseases, road traffic accidents, and reduced productivity (2).

In Saudi Arabia, insomnia is a common yet underaddressed condition. Recent studies have shown alarmingly high rates of insomnia symptoms in different segments of the Saudi population. For example, a large cross-sectional study found that nearly 63.9% of Saudis experienced symptoms consistent with insomnia and that students and young professionals were disproportionately affected (3). Another study in Riyadh City revealed that approximately one-third of adults had poor sleep quality, which is often associated with stress, poor lifestyle habits, and excessive use of electronic devices (4).

The management of insomnia often begins in community settings, especially because many individuals self-treat or seek over-the-counter (OTC) remedies before consulting a physician. Despite the availability of various pharmacological options—including sedating antihistamines, herbal remedies, melatonin, and prescription medications like benzodiazepines or non-benzodiazepine hypnotics—there is a considerable risk of misuse, dependency, and adverse effects when used without appropriate guidance (5). Non-pharmacological treatments, such as cognitive-behavioral therapy for insomnia (CBT-I), are considered the gold standard; however, access to such services remains limited in many countries, including Saudi Arabia.

Community pharmacists serve as the most accessible healthcare providers to the public and are frequently approached by patients seeking advice on minor ailments, including sleep problems. In this context, pharmacists play a crucial role in initial screening, providing safe OTC recommendations, offering lifestyle counseling, and referring patients to medical professionals when necessary. Research in other countries has demonstrated that pharmacist-led interventions can improve sleep outcomes and reduce inappropriate medication use (6). However, the actual practices of community pharmacists regarding insomnia management vary significantly and often depend on their training, knowledge, and workload.

In Saudi Arabia, the expanding role of pharmacists in public health and primary care is being increasingly recognized. However, there is a noticeable gap in the literature specifically examining how community pharmacists manage insomnia. While some studies have explored pharmacists’ knowledge and counseling practices in areas such as diabetes, hypertension, or smoking cessation, little is known about their role in sleep health. This lack of data poses a challenge to health planners and pharmacy educators seeking to design interventions that enhance pharmacists’ contributions to insomnia care. This study sought to fill this gap by assessing the everyday practices of community pharmacists in managing insomnia across three regions of Saudi Arabia during actual patient encounters using an SP approach. This was designed based on patient-centered pharmaceutical care, emphasizing comprehensive assessment, history taking, behavior, patient counseling, patient safety, and evidence-based decision-making. These points are in line with the quality primary care framework, which defines high-quality patient care and emphasizes safety, effectiveness, and a patient-centered approach.

## 2 Materials and methods

### 2.1 Study design

This study adopted a cross-sectional observational design utilizing SP to evaluate the everyday management of insomnia by community pharmacists. The SP approach is ideal for such assessments because it minimizes observer bias and reveals actual rather than self-reported practices (7). Each SP followed a standardized scenario to present a consistent clinical scenario across all pharmacy visits. The SP approach, also known in the literature as the mystery shopping technique, is a validated method for assessing professional practices and behaviors. It is a well-established and widely recognized instrument for evaluating the professional practices of community pharmacists (8).

This study was shaped by Donabedian’s Quality of Care Framework, which offers a practical way to understand how care is delivered in real-world settings (9). We considered the “structure” of community pharmacies, such as whether they were located in urban, suburban, or rural areas, and whether they operated as independent stores or part of local or national chains, as the context in which care takes place. The SP visits captured the “process” of care by observing how pharmacists actually managed a case of insomnia during routine practice. Using this framework helped us make sense of how the everyday realities of different pharmacy environments may influence the quality and consistency of care that patients receive.

High-quality insomnia management in community pharmacies is defined as.

1. Proper history taking, including sleep patterns, duration, triggers, symptoms, and chronic conditions.
2. Providing safety counseling.
3. Providing evidence-based non-pharmacological advice.
4. Referral to a physician whenever required (9), where each measured behavior was mapped into different parts of quality care.

Clinical assessment or history-taking quality, patient safety through patient counseling, evidence-based non-pharmacological counseling, patient-centeredness through professionalism, and providing referrals to physicians if necessary. Thus, high-quality insomnia management is explicitly framed within Donabedian’s process of care model, along with specific components defined based on clinical practice standards.

### 2.2 Study setting and subjects

This study employed a non-probability sampling approach, specifically purposive sampling combined with convenience selection, to recruit community pharmacies from the three selected regions. The decision to use this method was based on practical considerations and the study’s primary aim of exploring patterns of pharmacy practice rather than achieving statistical generalizability. Pharmacies were selected based on their accessibility during the data collection period while ensuring the inclusion of diverse practice settings, including different pharmacy types (independent vs. chain pharmacies) and locations (urban, semi-urban, and rural). A total of 251 pharmacies were visited and included in the analysis of an estimated 2,255 operating pharmacies in the three regions, accounting for more than 10% of the pharmacies visited. This approach enabled efficient data collection and ensured adequate representation of routine practice environments, aligning with the exploratory nature of simulated-patient research (10).

### 2.3 Study tool development and validation

A scenario was developed for SPs and included the following elements: a standardized participant presenting with short-term difficulty falling asleep over the past 7–10 days, without any history of chronic disease, medication use, or psychiatric illness, seeking “something to help sleep better,” without specifically asking for a drug or with a neutral demeanor, and limited motivation to assess pharmacist initiative was used across all visits to maintain consistency.

The scenario and data collection tools were developed in consultation with two practicing pharmacists. A structured checklist was developed to record all observed pharmacist behaviors, including history-taking items, safety counseling, non-pharmacological advice, communication behaviors, and referral practices. The authors developed a structured data collection checklist that was used by SPs to record all observed pharmacist behaviors (Table 1). The structured research tool’s content and face validity were developed by two faculty members from the College of Pharmacy at Al-Baha University. The checklist was refined following six pilot visits to pharmacies, ensuring clarity and consistency.

**Table 1:** The descriptive statistics of the pharmacist consultation process.

Variable	Sub variable	Frequency	Percentage
Duration of pharmacist interaction	1 min	63	25.1
	1.5 min	73	29.1
	2 min	54	21.5
	2.5 min	23	9.2
	3 min	6	2.4
	3.5 min	4	1.6
	4 min	1	0.4
Did the pharmacist ask about patterns of sleep difficulties?	Yes	139	55.4
	No	112	44.6
Did the pharmacist ask about the duration of sleep problems?	Yes	113	45.0
	No	138	55.0
Did the pharmacist ask if there were any triggers for sleep problems (including medical, psychological, lifestyle, behavioral, environmental, and routine)?	Yes	128	51.0
	No	123	49.0
Did the pharmacist ask about other symptoms accompanying your sleeplessness?	Yes	44	17.5
	No	207	82.5
Did the pharmacist ask about any actions you had taken?	Yes	71	28.3
	No	180	71.7
Did the pharmacist ask about the presence of chronic health problems?	Yes	46	18.3
	No	205	81.7

All SPs underwent intensive training to ensure uniformity and realism. SPs memorized the script and participated in role-playing exercises, and two piloting visits were conducted by each SP to ensure consistency. The SPs completed the structured checklist immediately after each encounter to minimize recall bias and ensure accurate documentation.

Following a pilot phase involving data collection from six pharmacies, minor amendments were made to the data collection form. These modifications included adding the time of the pharmacy visit (morning, evening, or night shift); grouping the triggers of insomnia into several categories (medical, psychological, lifestyle, behavioral, environmental, and changes in usual routine); and specifying whether the supplied product was currently registered by the Saudi Food and Drug Authority as a prescription, non-prescription medication, or herbal product.

### 2.4 Description of the structured checklist

A comprehensive structured checklist was designed for the SPs to record pharmacists’ responses relevant to insomnia management. It is divided into various sections, such as region of location, pharmacy type, location of pharmacies, time and day of visit, and dispensary load, along with the pharmacist’s demographics, such as estimated age, gender, and nationality. The consultation process documents the duration of interactions, including the key history-taking question, associated triggers, duration of symptoms, potential triggers, prior action taken, and the chronic health condition of the patient related to sleep disorders. The checklist also includes the pharmacist’s actions, such as ‘OTC product supply, the type of product with the name and strength, extent of dose information provided, administrations, side effects, warnings, and onset of action.’ Non-pharmacological advice given was recorded via whether the pharmacists discussed evidence-based sleep hygiene strategies such as ‘caffeine reduction, sleep scheduling, relaxation techniques, and limiting screen time.’ Pharmacists’ professionalism and communication were assessed through items evaluating self-introduction, professional appearance, privacy assurance, clarity of explanation, avoidance of complex jargon, checking patient understanding, patient preference considerations, and offering follow-up advice regarding the time of seeking medical advice. Finally, an open-ended section allowed the SP to document additional requirements or observations. The checklist was developed by the research team members, i.e., two faculty members, for face and content validity and refined to ensure clarity, consistency, and usability.

### 2.5 Data collection

Data were collected by three pharmacy interns through face-to-face interactions with community pharmacists. Each pharmacy was visited once, regardless of the number of pharmacists working in different shifts. The data collection form was completed immediately after leaving the pharmacy to ensure that the observation activity remained unnoticed by the pharmacy staff. Each SP measured the duration of the consultation using a mobile phone timer kept discreetly in their pocket. The timer was started when the pharmacist began speaking and stopped when the conversation ended. The SPs were instructed to remain calm and polite, allowing the pharmacist to lead the discussion and providing additional information only when prompted. If the pharmacist recommended a product, the SP continued the scenario up to the point of purchase before responding that they either already had the product at home and would use it or preferred to consult a physician first (11). Even though a composite quality index could be constructed, the current study treated each component related to insomnia management at community pharmacies, i.e., history-taking completeness, safety counseling, non-pharmacological advice, and physician referrals, as an individual domain of care. This approach may preserve the clinical meaning of each domain and avoid masking important differences between quality dimensions.

### 2.6 Statistical analysis

The data obtained from the survey were transformed into a Microsoft Excel spreadsheet, coded as required, and saved as a .csv file. The codes listed in the file were marked for the convenience of data processing and uploaded to the Statistical Package for Social Sciences (SPSS version 26; IBM, Armonk, NY, USA) for statistical analysis. The descriptive values, such as frequencies and percentages of the data, are shown in Tables 2–4. The chi-square (*χ*^2^) test was used to identify the association between the variables (dependent and independent). Tables 5 and 6 and Pearson’s correlation (*r*) were employed to identify the type of association among the variables chosen, which is shown in Table 7. Significant values were defined as *p* ≤ 0.05, highly significant values as *p* ≤ 0.01, and very highly significant values as *p* < 0.001. Pearson’s correlation was used to study the direction of the association of the variables for a better understanding. Correlation values of *r* = 1–3 were considered weak, 3–7 moderate, and 7–9.9 were considered strong (Tables 7 and 8) (12, 13). Binary logistic regression was performed to determine the predictors of pharmacists’ history taking and patient counseling (Table 8).

**Table 2:** The pharmacist demographics and pharmacy visit-related characteristics.

Variable	Sub variable	Frequency	Percentage
The region in which the pharmacy is located	Makkah	92	36.7
	Jazan	107	42.6
	Al-Baha	52	20.7
Location of pharmacy	Urban Area	109	43.4
	Suburban Area	124	49.4
	Rural	18	7.2
Type of pharmacy	National Chain	120	47.8
	Local Chain	65	25.9
	Independent	66	26.3
Time of visit	Morning shift (8:00 a.m. - 3:00 p.m.)	58	23.1
	Evening shift (3:00 p.m. to 11:00 p.m.)	155	61.8
	Night shift (11:00 p.m. to 7:00 a.m.)	38	15.1
Day of visit	Weekday	154	61.4
	Weekend	97	38.6
Dispensary load (number of patients at the time of visit)	Quiet (zero customers)	139	55.4
	Low (1 or 2 customers)	82	32.7
	Moderate (3 to 5 customers)	17	6.8
	Busy (more than 5 customers)	13	5.2
Estimated age of pharmacist	Less than 30 years	85	33.9
	30–40 Years	121	48.2
	More than 40 years	45	17.9
Gender of pharmacist	Male	235	93.6
	Female	16	6.4
Nationality of pharmacist	Saudi	44	17.5
	Non-Saudi	207	82.5

**Table 3:** The descriptive statistics of the pharmacist action process.

Variable	Sub variable	Frequency	Percentage
What actions did the pharmacist perform?	Supplied an OTC product	227	90.4
	Supplied an OTC product and provided non-pharmacological advice	17	6.8
	Provided non-pharmacological advice	7	2.8
If a product was provided, specify the name and strength of the product.	None	6	2.4
	Melatonin (1–5 mg)	162	64.5
	MEL + PCT (500 mg) + DPH (25 mg)	4	1.6
	MEL + Ashwagandha (300 mg)	3	1.2
	Valerian root (120 mg) + Melissa leaf (80 mg)	12	4.8
	Magnesium pills	4	1.6
	Strep wills night water or syrup	3	1.2
	Chlorpheniramine	1	0.4
	PCT + DPH	44	17.5
	Ashwagandha	2	0.8
	MEL + Melissa	2	0.8
	Primrose oil	1	0.4
	MEL + Valerian	1	0.4
	DPH + Dextromethorphan	1	0.4
	Dream water	3	1.2
	Valerian	1	0.4
	MEL + St. John’s wort root	1	0.4
What information did the pharmacist provide about the products?	Generic name	95	42.2%
	Administration times (when and how often to take it)	92	40.9%
	Brand name	81	36.0%
	Dosage	47	20.9%
	Dosage form (e.g., tablet, capsule, syrup)	47	20.9%
	Side effects and warnings	11	4.9%
	Time needed for the medication to take effect	3	1.3%
	Maximum recommended duration of use	2	0.9%
If non-pharmacological advice was provided, which of the following topics were addressed?	Reducing caffeine intake, especially in the evening	78	32.4%
	Limiting screen time before bed	25	10.4%
	Establishing a regular sleep schedule	20	8.3%
	Creating a comfortable sleep environment (dark, quiet, cool)	15	6.2%
	Going to bed only when sleepy	12	5.0%
	Avoiding heavy meals before bedtime	10	4.2%
	Avoiding naps late in the day	9	3.7%
	Using relaxation techniques (.)	6	2.5%
	None/Empty/N/A/Non	78	32.4%
	Other (exercise, marriage)	2	0.8%

MEL, Melatonin; PCT, Paracetamol; DPH, Diphenhydramine.

**Table 4:** The descriptive statistics of pharmacists’ professionalism, communication, and interaction.

Variable	Sub variable	Frequency	Percentage
Did the pharmacist introduce himself/herself?	Yes	38	15.1
	No	213	84.9
Did the pharmacist have a professional appearance (e.g., lab coat, name badge)?	Yes	215	85.7
	No	36	14.3
Was your privacy ensured during the consultations?	Yes	120	47.8
	No	131	52.2
Did the pharmacist explain the reason for asking the questions?	Yes	51	20.3
	No	200	79.7
Did the pharmacist avoid using inappropriate language (e.g., medical jargon or overly technical terms)?	Yes	240	95.6
	No	11	4.4
Did the pharmacist ask if you needed any additional information or had any questions?	Yes	39	15.5
	No	212	84.5
Did the pharmacist consider your preferences (e.g., medication choice, dosage forms)?	Yes	48	19.1
	No	203	80.9
Did the pharmacist check your understanding of the recommendations?	Yes	98	39.0
	No	153	61.0
Did the pharmacist offer a way to follow up (e.g., provide a phone number or invite you to return to the pharmacy)?	Yes	12	4.8
	No	239	95.2
Did the pharmacist tell you when you should visit a doctor?	Yes	15	6.0
	No	236	94.0

**Table 5:** Chi-square associations between pharmacy framework, pharmacist demographics, and consultation quality indicators.

Main variable	Associate variable	*χ*^2^-value	df	*p*-value	Significance
The region in which the pharmacy is located: Jazan, Makkah, Al-Baha	Did the pharmacist ask about patterns of sleep difficulties?	69.899	2	0.000	Extremely significant
	Did the pharmacist ask about the duration of sleep problems?	85.228	2	0.000	Extremely significant
	Did the pharmacist ask if there were any triggers for sleep problems?	101.994	2	0.000	Extremely significant
	Did the pharmacist ask about other symptoms accompanying your sleeplessness?	55.751	2	0.000	Extremely significant
	Did the pharmacist ask if you needed any additional information or had any questions?	43.407	2	0.000	Extremely significant
Pharmacy location:	Asked about the pattern of sleep difficulty	9.234	2	0.01	Highly significant
	Asked about triggers for the sleep problem	11.61	2	0.003	Highly significant

**Table 6:** Chi-square associations between type of pharmacy, time, and day of consultation, and quality indicators.

Main variable	Associate variable	*χ*^2^-value	df	*p*-value	Significance
Type of pharmacy (national chain, local chain, or independent)	Checked your understanding of recommendations	18.717	2	0.000	Extremely significant
Time of visit	Asked about the pattern of sleep difficulty	19.517	2	0.000	Extremely significant
	Asked about the duration of the sleep problem	21.049	2	0.000	Extremely significant
	Asked about other symptoms	19.2	2	0.000	Extremely significant
	Was your privacy ensured	27.099	2	0.000	Extremely significant
	Told you when to visit a doctor	15.7	2	0.000	Extremely significant
Gender of pharmacist	Did the pharmacist introduce himself/herself	38.231	1	0.000	Extremely significant
Nationality of pharmacist	Did the pharmacist introduce himself/herself	18.707	1	0.000	Extremely significant
Duration of interaction	Asked about the pattern of sleep difficulty	60.244	12	0.000	Extremely significant
	Asked about the duration of the sleep problem	42.918	12	0.000	Extremely significant
	Asked about other symptoms	35.835	12	0.000	Extremely significant
	Product name and strength provided	778.701	192	0.000	Extremely significant

**Table 7:** Correlation analysis of pharmacist consultation quality indicators regarding sleep-related issues.

Variable 1	Variable 2	*R*-value	*p*-value	Interpretation
Did the pharmacist ask about patterns of sleep difficulties?	Did the pharmacist ask about the duration of sleep problems?	0.506	0.000	Moderate positive correlation
	Did the pharmacist ask if there were any triggers for sleep problems?	0.531	0.000	Moderate positive correlation
	Did the pharmacist check your understanding of the recommendations?	0.505	0.000	Moderate positive correlation
Did the pharmacist ask about the duration of sleep problems?	Did the pharmacist ask if there were any triggers for sleep problems?	0.471	0.000	Moderate positive correlation
	Did the pharmacist check your understanding of the recommendations?	0.507	0.000	Moderate positive correlation
Did the pharmacist ask if there were any triggers for sleep problems?	Did the pharmacist check your understanding of the recommendations?	0.539	0.000	Moderate positive correlation
Did the pharmacist ask about other symptoms accompanying your sleeplessness?	Did the pharmacist ask about any actions you had taken (if any)?	0.176	0.005	Weak positive correlation
	Did the pharmacist ask about the presence of any chronic health problems?	0.296	0.000	Weak positive correlation
	Did the pharmacist explain the reason for asking the questions?	0.236	0.000	Weak positive correlation

**Table 8:** The logistic regression summary of the determinants of patient history taking and patient counseling.

Dependent variable	Predictor variable	*p*-value	Odds ratio	Interpretation
History taking: pattern of sleep difficulty	Dispensary load (number of customers)	0.043	1.883	More likely
	Duration of interaction	0.007	0.338	Less likely
	Asking about triggers	0.018	3.154	Strong increase
	Providing non-pharmacological advice	0.003	0.101	Much less
History taking: duration of sleep problem	Day of visit	0.000	509.979	Very strong
	Asking about accompanying symptoms	0.000	6567.348	Extremely high
	Asking about chronic health problems	0.000	941.751	Very high
	Asking if additional info is needed	0.002	410.669	Strong increase
	Professional appearance	0.045	9.396	Higher odds
History taking: asking about triggers	Type of pharmacy	0.010	0.000	Almost none
	Day of visit	0.006	1.39E+15	Extremely high
	Gender of the pharmacist	0.025	2.136	Higher odds
	Duration of interaction	<0.001	3.87E+17	Extremely high
	Privacy ensured	<0.001	0.386	Less likely
History taking: asking about symptoms	Duration of interaction	0.007	0.000	Almost none
	Asking about triggers	<0.001	2.78E+37	Extremely high
	Considering patient preferences	0.010	5,683,665.40	Very high
	Checking Understanding	<0.001	1,945,599.82	Very high
History taking: asking about actions taken	Asking about sleep patterns	0.002	0.000	Almost none
	Asking about symptoms	<0.001	0.000	Almost none
	Product provided (if any)	0.049	0.001	Very low
	Explaining the reason for questions	0.002	1.20E+27	Extremely high
History taking: chronic health problems	Duration of sleep problem	0.025	–	Not estimable
	Asking about symptoms	0.004	–	Not estimable
	Asking about actions taken	0.021	–	Not estimable
	Asking if additional info needed	0.034	–	Not estimable
Safety advice given	Dispensary load	0.002	0.040	Much less
	Asking about the pattern of sleep difficulty	0.010	57.647	Strong increase
	Privacy ensured	0.045	144.092	Very high
	Explaining the reason for questions	0.022	1,374.144	Extremely high
Non-pharmacological advice given	Type of pharmacy	0.032	145.669	Very high
	Time of visit	0.029	125,577.590	Extremely high
Doctor referral given	Pharmacy location	0.014	373.404	Very high
	Time of visit	0.011	0.014	Much less
	Day of visit	0.012	177,700.538	Extremely high
	Offering a way to follow up	0.028	55.035	Strong increase

Several history-taking models (triggers, symptoms, actions taken) reached maximum iterations or detected a perfect fit, reporting *p* < 0.001 for most included variables. ‘–’ indicates the odds ratio cannot be computed.

### 2.7 Ethical considerations

Ethical approval was obtained from the Al-Baha University Institutional Review Board (IRB). Pharmacists were not informed beforehand to preserve authenticity, but the study complied with the ethical guidelines for simulated encounters (7). Pharmacy identities remained anonymous, and the results were reported only in aggregate.

## 3 Results

A total of 251 pharmacy visits were conducted across three regions of Saudi Arabia, i.e., Jazan, Makkah, and Al-Baha, of which the majority of respondents were in Jazan (42.6%) and suburban areas (49.4%), with national chain pharmacies being the most prevalent (47.8%). Patient visits primarily occurred during the evening shifts (61.8%) on weekdays (61.4%) when the dispensary had no customers (55.4%, zero customers). Pharmacists were mostly male (93.6%), non-Saudi (82.5%), and 30–40 years old (48.2%) (Table 2).

The median minimum duration of pharmacist interaction with the visitor was 1.5 min (median interquartile range: 1–2 min), with most consultations being concluded within 1–2 min (75.7%). Pharmacists most frequently inquired about the pattern of sleep difficulty in the majority of cases (55.4%) and the triggers responsible for the sleep problem (51.0%), including medical, psychological, lifestyle/behavioral, environmental, and lifestyle changes. Inquiries about the duration of the sleep problem occurred in 45.0% of consultations, indicating that duration is a commonly assessed factor. Less commonly asked questions were about other associated symptoms (17.5%), whether any actions had already been taken (28.3%), and the presence of chronic health problems (18.3%) (Table 1).

In most consultations that took place (90.4%, *n* = 227), pharmacists mainly provided an over-the-counter (OTC) product on their own. A smaller number of consultations included both providing an OTC product and non-pharmacologic advice (6.8%, *n* = 17); however, an even smaller number of consultations involved no OTC product being dispensed (2.8%, *n* = 7). Of the total consultations (*n* = 245) in which a product was dispensed, melatonin (1–5 mg) was prescribed the most often (64.5%, *n* = 162), followed by a combination of diphenhydramine-paracetamol (17.5%, *n* = 44), and all the rest were prescribed less than 10% of the time (Table 3).

Among all cases in which patients were given some type of information about their prescription products (*n* = 225), pharmacists reported the most frequent types of information they gave as the generic name for the product (42.2%) and how it should be administered (40.9%), although they were least likely to provide information on dosage (20.9%). Critical safety information was rarely communicated: side effects/warnings (4.9%), time to effect (1.3%), and maximum duration of use (0.9%).

Additionally, Non-pharmacological advice was provided only in 24 cases (approximately 9.6% of patient interactions). The most common recommendations included reducing coffee/caffeine consumption, reducing screen time, and establishing a consistent sleep schedule. In addition to this limited mention of several other evidence-based methods, no recommendation was made in nearly one-third of visits (32.4%) (Table 3).

Pharmacists largely displayed a professional image (85.7%) and used proper language (95.6%) when interacting with patients; however, important patient-centered communication practices were deficient. In the majority of interactions (84.9%), there was little to no introduction by the pharmacist regarding who they were. Privacy was maintained in just over half (52.2%) of all patient-pharmacist communications.

Pharmacists generally performed poorly in terms of active engagement in communication. Fewer than one-fifth of the time (20.3%), pharmacists asked their patients additional questions during the interaction. Similarly, pharmacists invited patients to ask them additional questions approximately one-sixth of the time (15.5%) and considered the preference of patients regarding their own health or treatment about one-fifth of the time (19.1%). Patient understanding was assessed by pharmacists in nearly two-fifths (39.0%) of encounters. However, pharmacists offered follow-up information to patients about their treatment options infrequently (4.8%) and recommended that patients seek medical attention for themselves even less frequently (6.0%) (Table 4).

Chi-square analysis highlighted the significant association between pharmacy region and consultation quality indicators (Table 5). There was a significant relationship between many consultation quality indicators and other variables. Most notably, the pharmacy region affected how pharmacists performed history-taking activities and interacted with patients regarding their private information (*p* ≤ 0.001).

Pharmacy site and pharmacy type both affected certain aspects of the quality of consultations, such as history taking and patient comprehension (*p* < 0.01).

The time of the visit and the length of the interaction significantly affected the amount of information taken from each patient to identify symptoms or assure the patient’s privacy (p ≤ 0.001), while pharmacist demographics, such as sex and country of origin, were related to the manner in which the pharmacist introduced themselves during the interaction. A full list of all the statistics is shown in Tables 5 and 6.

Correlation analysis revealed many relationships to be statistically significant but also very weak. The regional variation for privacy assurance had a moderate correlation (*r* = −0.605; *p* < 0.001). Interaction time was correlated with several of the consultation elements (Pattern Inquiry *r* = −0.250, *p* < 0.0001, Symptom Assessment *r* = −0.219, *p* < 0.0001); interaction time influenced the extent of information gathering.

There were strong positive correlations between the elements involved in gathering history, such as Pattern-Trigger (*r* = 0.531, *p* < 0.0001), Duration-Pattern (*r* = 0.506, *p* < 0.0001), Triggers-Duration (*r* = 0.471, *p* < 0.0001), and Patient Understanding (*r* = −0.539, *p* < 0.0001) (Table 7) (Supplementary material 1).

Binary logistic regression analysis highlighted several important factors that influence pharmacists’ clinical performance, where dispensing load transpired as a consistently significant predictor, negatively influencing both history-taking patterns (*p* = 0.043) and provision of safety advice (*p* = 0.002). History taking was strongly associated with the duration of interaction, particularly with the duration of sleep problems (*p* = 0.001). Furthermore, the pharmacists who inquired regarding the triggers for sleeplessness were three times more likely to detect the pattern of sleep difficulty (OR = 3.154; *p* = 0.018), and triggers, accompanying symptoms, prior actions taken, and chronic health problems were identified as statistically significant (Omnibus tests, *p* < 0.01). These variables can be considered strongly linked to the results, as the model reached a perfect fit and these variables were classified as strong predictors of the outcomes. In particular, the duration of the interaction was significant for both trigger inquiry (*p* < 0.001) and symptoms (*p* = 0.007). Clinical communication covering sleep patterns was significantly associated with asking about triggers (*p* < 0.001), symptoms (*p* < 0.001), and prior action taken (*p* = 0.002). Additionally, professional factors, such as ensuring patients’ privacy (*p* < 0.001) and checking patients’ understanding (*p* < 0.001), resulted in a higher quality of history-taking across all categories. Privacy during consultations significantly increased the likelihood of providing safety advice (OR = 144.092; *p* = 0.045) and assessing detailed issues related to sleep duration (*p* = 0.037). Pharmacy location, time of visit, and day of visit significantly predicted the likelihood of both doctor referrals and provision of non-pharmacological advice.

However, the gender of the pharmacist was consistently insignificant in predicting the likelihood of asking about sleep patterns (*p* = 0.808), providing safety advice (*p* = 0.997), or providing non-pharmacological interventions (*p* = 1.000). The pharmacy location did not highlight the quality of sleep pattern determination (*p* = 0.564) or the issuing of safety advice (*p* = 0.713). Professional behaviors such as self-introduction (*p* = 0.231; for pattern assessment) and patient preferences (*p* = 0.997 for pattern assessment and *p* = 0.723 in non-drug recommendations) were also identified as insignificant. Even the product-provided attribute to the patient failed to identify whether a pharmacist would ask about sleep patterns (*p* = 0.893) or provide safety advice (*p* = 0.472).

Non-pharmacological advice was provided in 132 cases (either alone or together with an OTC sleep medication). However, it was the sole recommendation in only 24 cases (approximately 9.6% of patient interactions), of which reducing caffeine intake in the evening was the most frequently recommended (77.3%). Other commonly advised strategies included limiting screen time before bed (17.4%), maintaining a regular sleep schedule (15.2%), and creating a comfortable sleep environment (12.9%). Less common advice provided was reaching bed only if feeling sleepy (9.1%), avoiding heavy meals during bedtime (7.6%), avoiding short naps (7.6%), and using relaxing techniques such as deep breathing and meditation (4.5%). The data highlighted that only a few core sleep hygiene practices were consistently emphasized, and several important evidence-based behavioral recommendations were focused far less on (Table 8; Supplementary material 2).

## 4 Discussion

This cross-sectional study employed SP visits to evaluate the real-world practices of community pharmacists in selected regions of Saudi Arabia, where pharmacists manage cases of short-term insomnia. The study outcomes highlight several strengths but are overshadowed by significant gaps in patient assessment, counseling, and referrals, highlighting the important areas of systematic intervention and professional development of pharmacists. The current study examined and analyzed various behavioral domains of community pharmacists that reflect the quality of insomnia management. These domains were analyzed separately because each represents a unique and clinically relevant dimension of the quality of care for insomnia. Combining them into a single index may decrease the impact of individual domain outcomes and may fail to identify variations in practice, especially in areas such as safety counseling and physician referrals, which have different implications for patient outcomes. This multidimensional approach parallels the patient-centered pharmaceutical care framework, which views quality as the sum of several distinct but interrelated behavioral outcomes.

### 4.1 Key findings of the study

The most crucial findings of the study identified the overwhelming over-the-counter (OTC) product-centered management of insomnia, i.e., melatonin (64.5%) at different doses by pharmacists aged above 30 years, which aligns with a study published that explains a similar attitude by a community pharmacist choosing melatonin as OTC management medication in sleep management (14). This indicates a general awareness of common products with safety as a priority over traditional sedative antihistamines. However, the consultations were brief, with a median time of 1.5 min per patient and without complete history taking. A similar study published expresses a pharmacist consultation time of 2 min (ranging between 0.2 and 4 min) and a combination of paracetamol and diphenhydramine was the most frequently dispensed, followed by valerian and lemon balm as a novel formulation (10). In the majority of cases, the questions related to sleep patterns (55.4%) and triggers (51%) were relatively common, and critical assessments including the chronic health problems (18.3%) and other relative symptoms (17.5%), were completely omitted in most of the cases, including prior actions taken by the patients, if any, which were missed in 71.7% of interactions. Similarly, a study explained that the community pharmacist did not provide any information related to the adverse drug reaction or drug interactions, and no information related to the patient’s past medication or medical history was assessed before providing the sleep management (10) which aligns with the current study. Furthermore, patient counseling was also limited and did not include critical safety information, such as side effects, duration of use, and drug interactions, which was provided in less than 5% of cases, and very important non-pharmacological advice related to insomnia management was offered in 9.6% of cases. Pharmacists’ communication and professional practices were inconsistent, with a low rate of self-introduction, privacy assurance (47.8%), checking patients’ understanding (39%), and advising physicians (6%). If any information related to the drugs was provided, it was the generic name (42.2%) and administration time (40.9%), highlighting an opportunity for improvement. On average, 11.6% of the products dispensed for the management of sleep disorders are from plant sources, and knowledge regarding the adverse drug reactions and drug interactions of these products needs to be incorporated with formal training of pharmacists (15).

### 4.2 Interpretation and implication

This study highlights that community pharmacists in Saudi Arabia are primarily focused on dispensing OTC sleep medications or aids rather than functioning as comprehensive healthcare providers for the management of insomnia, similar to a study published by Alhussain et al. (16). The melatonin, which was provided at a high rate, aligns with international guidelines, favoring older antihistamines for short-term use. However, complete reliance on the pharmacological solution and the lack of knowledge on sleep hygiene and related issues counseling, including reducing caffeine and screen time, may represent a missed opportunity for effective care that addresses the behavioral causes, as the improper use of OTC medications can lead to untoward outcomes (17). The shortness of proper consultation and lack of safety counseling raise concerns about the potential for misuse of sleep medications along with delayed diagnosis of serious comorbidities like anxiety, sleep apnea, stress, and patient dissatisfaction (18).

The regional variations in almost all aspects of pharmacists’ practice, including history taking, communication, and referrals, are among the most prominent and concerning results. These disparities suggest either a lack of standardized national protocols or sleep health training, leading to inconsistent and potentially unbalanced care depending on the patient’s location, or they indicate that guidelines are not followed consistently. Better knowledge, attitude, and practice guidelines or workshops can develop a positive attitude toward professional behavior, bypassing geographical and knowledge disparities (19). The association between longer consultation duration and more thorough assessment practices underscores time pressure, which can be considered a major barrier to optimal care. This is supported by the finding that a higher dispensary load negatively affects privacy and professional appearance (20).

The demographic-based correlation, such as female pharmacists and Saudi nationals, demonstrating better communication and assessment practices in a few domains. This points to the potential influence of proper training, cultural communication style, and job stability—factors that cannot be neglected. However, further qualitative investigation into these domains is warranted (21). The study also identified that national chain pharmacies showed a positive association with study domains such as professional appearance, checking and understanding, and counseling, suggesting that corporate training and protocols can have a beneficial impact. However, this effect was not uniform across all quality indicators (22). In the era of artificial intelligence (AI), current and future community pharmacist training can be designed by employing AI tools, following the study published by Easwaran et al., where 85.5% of participants agreed to benefit from such training (23).

Various system-level barriers appear to contribute to the limited patient counseling, incomplete history taking, and low physician referral rate. Certain factors, such as a high dispensing workload, short consultation time, and improper workflow design, which prioritize drug supply over clinical interaction, restrict pharmacists’ ability to conduct a complete patient assessment. Training gaps in sleep health-related issues and inconsistencies in national protocols further contribute to variability in practice. These issues may explain why highly motivated and knowledgeable pharmacists struggle to deliver optimal insomnia care.

Binary logistic regression analysis highlighted the operational and environmental factors, which are the primary independent variables of the quality of care provided in community pharmacies for sleep-related disorders. The significance of dispensing load indicates that high patient visits may force pharmacists to shorten clinical interactions, leading to decreased safety counseling and comprehensive history taking. This is further supported by the data showing that a longer duration of interactions correlates with more detailed clinical inquiries. The clinical perception remains narrow, where the advice provided by the pharmacist is dominated by caffeine reduction, whereas evidence-based behavioral strategies, such as relaxation techniques, are rarely discussed. The strong link between privacy and safety advice indicates that a professional consultation setting is not merely a formality but a quality requirement for better clinical outcomes. Factors such as pharmacists’ gender and region were largely insignificant; interventions to improve practice must focus on workload management and standardizing sleep hygiene protocols, ensuring that all critical lifestyle triggers are addressed. High-quality history taking is not a series of isolated events but a unified clinical behavior; hence, a perfect fit was observed. If a pharmacist is engaged in professional consultation practice, they are almost statistically certain of comprehensive history taking. The time of interaction is a critical resource, as it has resulted in significant outcomes, where shorter interactions may force a pharmacist to bypass detailed inquiries into triggers and symptoms. Furthermore, the medication being dispensed may be influenced by the history-taking procedure, where a strong link between inquiries regarding prior actions taken and the product provided is well established. Irrespective of the nationality of pharmacists and the location of pharmacies, the importance of communication skills demonstrates providing better patient-centered care based on standardized consultation protocols. These findings highlight the need for targeted training emphasizing the clinical interaction between various history-taking steps to ensure that no critical information is neglected.

The crucial domains of community pharmacists, such as poor counseling and minimal referral behavior, have important implications for national pharmacy policy and workforce development. Strengthening sleep health training, including standardized consultation protocols and redesigning workflows to protect clinical presentation, may enhance the quality of insomnia management. National guidelines that emphasize non-pharmacological interventions, safety counseling, and timely referrals may help shift practice from drug dispensing to patient-centered care. These findings highlight the need for system-level reforms rather than solely focusing on individual pharmacists’ performance at the community pharmacy level.

### 4.3 Influence of demographics and operational factors in pharmacists’ practice

Dispensing workload, interaction duration, and pharmacy characteristics are the most important predictors of the quality of pharmacist-patient interactions. Dispensing workload significantly influences the quality of pharmacist-patient interactions; the amount of work a pharmacist has to do (i.e., how many prescriptions they have to fill) will determine their ability to take a full medical history and provide adequate counseling to patients. The greater the number of prescriptions that need to be filled, the less likely it is that a complete medical history can be taken and appropriate medication counseling provided. This is consistent with prior research showing that workflow constraints in community pharmacies result in product provision rather than patient-centered care (22).

Interaction duration was also a significant predictor of the quality of pharmacist-patient interactions; the length of the consultation had a significant positive association with the quality of the assessment of sleep patterns, triggers, and symptoms. This indicates that there is value in having sufficient time to provide quality care. It appears that shorter consultations may result in some clinical information being missed during the assessments.

There were also variations observed in terms of pharmacy type; chain pharmacies generally performed well in all aspects examined, although it should be noted that this could reflect a combination of standardization through standardized operating procedures and corporate-based staff training. However, regional differences in practice were also identified, particularly with regard to privacy assurance and communication behaviors, which suggests a lack of consistency in service delivery that may be related to differences in training, supervision, or adherence to national or local guidelines.

Temporal factors, such as time and day of visit, also appeared to affect the way pharmacists practiced. Certain times/days of consultation have been shown to be related to enhanced interaction quality. This may be due to differences in workload and/or staffing patterns at different times/days.

Gender and nationality were the two most common pharmacist-related characteristics investigated in relation to practice outcomes. These were shown to have little to no effect and therefore indicate that system-level factors are likely more influential than individual attributes.

These findings support the idea that performance is not solely dependent upon individual competence but is heavily affected by a variety of environmental/organizational/workflow-related factors.

### 4.4 Strengths and limitations

The current study employed the SP methodology, which provides an objective, unbiased assessment of actual practice and pharmacist behavior, further avoiding the social desirability bias associated with self-reported surveys. Standardized scenarios, multi-region sampling, and centered sampling increase the validity and generalizability of the findings within the Saudi Arabian context.

Several limitations must be acknowledged, such as the selection of non-probabilistic sampling, as the results may not represent all community pharmacies in Saudi Arabia. The use of straightforward scenarios involving single patients may not explain how pharmacists manage more complex cases, such as those involving older adult patients, chronic insomnia patients, or patients with suspected comorbidities. This study measured the recommendations, advice, and products meant for the management of sleep disorders but did not assess the clinical appropriateness and accuracy of every recommendation made. Further thematic analysis in future research related to a similar topic would benefit the outcomes (24, 25). Although the SP methodology is designed to minimize the Hawthorne effect by concealing the assessment from the participants, it cannot eliminate all possibilities of the Hawthorne effect. Consequently, there could be the possibility that the pharmacists who participated would be able to detect an assessment (i.e., they would “know” when they were being evaluated) and, therefore, might alter their behavior based on contextual cues, which may improve their performance in specific areas of consultation, thus overestimating the quality of usual practice. Including a large sample size from multiple regions and pharmacy types may enhance the outcomes of the study, would strengthen the understanding of insomnia-related issues—an important yet unexplored area—and may provide valuable suggestions for updating national policies through pharmacy practice. Although the SPs completed the checklist immediately after each interaction, they stated that they did not have difficulty recalling their interactions while the simulation was taking place. There is still a chance of recall bias because the average time per consultation was very low (1.2 min) and the checklist was lengthy.

### 4.5 Recommendations

Based on the study findings, the following recommendations have been developed for implementation. These include: (1) education and training of pharmacists through continuous professional development (CPD) programs focusing on evidence-based insomnia management; and (2) improving patient assessment and counseling skills for OTC medications for sleep disorders.

To mitigate the regional disparities, the Saudi Ministry of Health and professional bodies like the Saudi pharmaceutical societies should develop and circulate clear clinical practice guidelines for community pharmacies regarding the management of common ailments such as insomnia.

Workflow optimization and proper pharmacy management should explore ways to reduce time pressure and dispensary load during peak hours for staff, including designating a counseling area to facilitate privacy and patient-centered interactions.

It is recommended to consider the incorporation of aspects of patient counseling and referrals into inspection criteria, which may help in incentivizing a shift from a purely dispensing to a more clinical role with a service-centered model.

## 5 Conclusion

The current study provides practice-based insights into how a community pharmacy in Saudi Arabia manages insomnia. Pharmacists demonstrated accessibility and preference for relatively safer OTC products, such as melatonin, in the majority of cases of insomnia; their current practice falls short of optimal patient-centered standards. Critical gaps in inclusive assessments, non-pharmacological counseling, safety communications, and a lack of appropriate referrals pose risks to patient safety and outcomes. Highly significant variation in practice quality based on region, pharmacy type, and workload highlights systemic rather than individual issues. Addressing these gaps through targeted education, standardized guidelines, and a supportive practice environment is essential to fully realize the potential of community pharmacists in improving sleep health and public health outcomes in Saudi Arabia.

## Data Availability

The raw data supporting the conclusions of this article will be made available by the authors, without undue reservation.

## Supplementary material

Supplementary Table 1 Detailed Pearson correlation analysis of pharmacist consultation quality indicators.

Supplementary Table 2 Detailed non-pharmacological advice provided by community pharmacists.
