# Supplementary material for: Evaluating community pharmacists’ practices in managing insomnia in Saudi Arabia: a cross-sectional study using simulated patient visits
Source: Front Public Health. 2026 Jun 16;14:1792148. doi: 10.3389/fpubh.2026.1792148 (PMC13326896; doi:10.3389/fpubh.2026.1792148)
Supplement: Supplementary Table 2 — Detailed non-pharmacological advice provided by community pharmacists. [file Supplementary_file_2.DOCX]

**Logistic Regression**

| **Case Processing Summary** | | | |
| --- | --- | --- | --- |
| Unweighted Cases^a^ | | N | Percent |
| Selected Cases | Included in Analysis | 234 | 93.2 |
|  | Missing Cases | 17 | 6.8 |
|  | Total | 251 | 100.0 |
| Unselected Cases | | 0 | .0 |
| Total | | 251 | 100.0 |

| a. If weight is in effect, see classification table for the total number of cases. |
| --- |

| **Dependent Variable Encoding** | |
| --- | --- |
| Original Value | Internal Value |
| No | 0 |
| Yes | 1 |

**Block 0: Beginning Block**

| **Iteration History^a,b,c^** | | | |
| --- | --- | --- | --- |
| Iteration | | -2 Log likelihood | Coefficients |
|  |  |  | Constant |
| Step 0 | 1 | 92.765 | -1.880 |
|  | 2 | 67.191 | -2.772 |
|  | 3 | 63.165 | -3.295 |
|  | 4 | 62.922 | -3.464 |
|  | 5 | 62.920 | -3.479 |
|  | 6 | 62.920 | -3.479 |

| a. Constant is included in the model. |
| --- |
| b. Initial -2 Log Likelihood: 62.920 |
| c. Estimation terminated at iteration number 6 because parameter estimates changed by less than .001. |

| **Classification Table^a,b^** | | | | | |
| --- | --- | --- | --- | --- | --- |
|  | Observed | | Predicted | | |
|  |  |  | Non drug recommendation | | Percentage Correct |
|  |  |  | No | Yes |  |
| Step 0 | Non drug recommendation | No | 227 | 0 | 100.0 |
|  |  | Yes | 7 | 0 | .0 |
|  | Overall Percentage | |  |  | 97.0 |

| a. Constant is included in the model. |
| --- |
| b. The cut value is .500 |

| **Variables in the Equation** | | | | | | | |
| --- | --- | --- | --- | --- | --- | --- | --- |
|  | | B | S.E. | Wald | df | Sig. | Exp(B) |
| Step 0 | Constant | -3.479 | .384 | 82.191 | 1 | .000 | .031 |

| **Variables not in the Equation** | | | | | |
| --- | --- | --- | --- | --- | --- |
|  | | | Score | df | Sig. |
| Step 0 | Variables | 1. The region in which the pharmacy is located | 4.257 | 1 | .039 |
|  |  | 2. The pharmacy is located in | .092 | 1 | .762 |
|  |  | 3. Type of pharmacy | .538 | 1 | .463 |
|  |  | 4. Time of Visit | 1.077 | 1 | .299 |
|  |  | 5. Day of Visit | .071 | 1 | .790 |
|  |  | 6. Dispensary Load (Number of customers at the time of visit) | .055 | 1 | .814 |
|  |  | 1. Estimated Age of the Pharmacist: | .294 | 1 | .588 |
|  |  | 2. Gender of the Pharmacist | .459 | 1 | .498 |
|  |  | 3. Nationality of the Pharmacist (if known): | .020 | 1 | .887 |
|  |  | 1. What was the duration of the interaction?  (Start your mobile timer when the pharmacist begins the conversation, and stop it when the interaction ends.)  _______ minutes  Put numbers only (e.g., 0.5, 1, 1.5, 2, 2.5,3 ... etc.). | 15.923 | 1 | .000 |
|  |  | 2. Did the pharmacist ask about the pattern of sleep difficulty? | .351 | 1 | .554 |
|  |  | 3. Did the pharmacist ask about the duration of the sleep problem? | .775 | 1 | .379 |
|  |  | 4. Did the pharmacist ask if there were any triggers for the sleep problem (including medical, psychological, lifestyle and behavioral, environmental, and changes in usual routine)? | .130 | 1 | .718 |
|  |  | 5. Did the pharmacist ask about other symptoms accompanying your sleeplessness? | .020 | 1 | .887 |
|  |  | 6. Did the pharmacist ask about any actions you have already taken (if any)? | 2.956 | 1 | .086 |
|  |  | 7. Did the pharmacist ask about the presence of any chronic health problems? | 1.533 | 1 | .216 |
|  |  | 2. If a product was provided, provide the name and the strength of the product. | .384 | 1 | .536 |
|  |  | @3safetyadvicegivenbypharmacistx | .040 | 1 | .841 |
|  |  | 1. Did the pharmacist introduce himself/herself? | .003 | 1 | .960 |
|  |  | 2. Did the pharmacist have a professional appearance (e.g., lab coat, name badge)? | 1.269 | 1 | .260 |
|  |  | 3. Was your privacy ensured during the consultation? | 3.260 | 1 | .071 |
|  |  | 4. Did the pharmacist explain the reason for asking questions? | 1.862 | 1 | .172 |
|  |  | 5. Did the pharmacist avoid using inappropriate language (e.g., medical jargon or overly technical terms)? | 1.480 | 1 | .224 |
|  |  | 6. Did the pharmacist ask if you needed any additional information or had any questions? | .000 | 1 | .989 |
|  |  | 7. Did the pharmacist consider your preferences (e.g., medication choice, 29 form)? | .096 | 1 | .756 |
|  |  | 8. Did the pharmacist check your understanding of the recommendations ? | 1.838 | 1 | .175 |
|  |  | 9. Did the pharmacist offer a way to follow up (e.g., provide a phone number or invite you to return to the pharmacy)? | .390 | 1 | .532 |
|  |  | 10. Did the pharmacist tell you when you should visit a doctor? | .424 | 1 | .515 |
|  | Overall Statistics | | 38.886 | 28 | .083 |

| **Omnibus Tests of Model Coefficients** | | | | |
| --- | --- | --- | --- | --- |
|  | | Chi-square | df | Sig. |
| Step 1 | Step | 48.436 | 28 | .010 |
|  | Block | 48.436 | 28 | .010 |
|  | Model | 48.436 | 28 | .010 |

| **Model Summary** | | | |
| --- | --- | --- | --- |
| Step | -2 Log likelihood | Cox & Snell R Square | Nagelkerke R Square |
| 1 | 14.484^a^ | .187 | .793 |

| a. Estimation terminated at iteration number 20 because maximum iterations has been reached. Final solution cannot be found. |
| --- |

| **Hosmer and Lemeshow Test** | | | |
| --- | --- | --- | --- |
| Step | Chi-square | df | Sig. |
| 1 | .011 | 8 | 1.000 |

| **Contingency Table for Hosmer and Lemeshow Test** | | | | | | |
| --- | --- | --- | --- | --- | --- | --- |
|  | | Non drug recommendation = No | | Non drug recommendation = Yes | | Total |
|  |  | Observed | Expected | Observed | Expected |  |
| Step 1 | 1 | 23 | 23.000 | 0 | .000 | 23 |
|  | 2 | 23 | 23.000 | 0 | .000 | 23 |
|  | 3 | 23 | 23.000 | 0 | .000 | 23 |
|  | 4 | 23 | 23.000 | 0 | .000 | 23 |
|  | 5 | 23 | 23.000 | 0 | .000 | 23 |
|  | 6 | 23 | 23.000 | 0 | .000 | 23 |
|  | 7 | 23 | 23.000 | 0 | .000 | 23 |
|  | 8 | 23 | 23.000 | 0 | .000 | 23 |
|  | 9 | 23 | 22.989 | 0 | .011 | 23 |
|  | 10 | 20 | 20.011 | 7 | 6.989 | 27 |

| **Classification Table^a^** | | | | | |
| --- | --- | --- | --- | --- | --- |
|  | Observed | | Predicted | | |
|  |  |  | Non drug recommendation | | Percentage Correct |
|  |  |  | No | Yes |  |
| Step 1 | Non drug recommendation | No | 226 | 1 | 99.6 |
|  |  | Yes | 3 | 4 | 57.1 |
|  | Overall Percentage | |  |  | 98.3 |

| a. The cut value is .500 |
| --- |

| **Variables in the Equation** | | | | | | | |
| --- | --- | --- | --- | --- | --- | --- | --- |
|  | | B | S.E. | Wald | df | Sig. | Exp(B) |
| Step 1^a^ | 1. The region in which the pharmacy is located | -10.533 | 1017.085 | .000 | 1 | .992 | .000 |
|  | 2. The pharmacy is located in | -8.273 | 8.010 | 1.067 | 1 | .302 | .000 |
|  | 3. Type of pharmacy | 4.981 | 2.322 | 4.601 | 1 | .032 | 145.669 |
|  | 4. Time of Visit | 11.741 | 5.393 | 4.739 | 1 | .029 | 125577.590 |
|  | 5. Day of Visit | .346 | 7.038 | .002 | 1 | .961 | 1.414 |
|  | 6. Dispensary Load (Number of customers at the time of visit) | 1.472 | 2.305 | .408 | 1 | .523 | 4.360 |
|  | 1. Estimated Age of the Pharmacist: | 4.893 | 2.511 | 3.797 | 1 | .051 | 133.361 |
|  | 2. Gender of the Pharmacist | -3.425 | 9321.581 | .000 | 1 | 1.000 | .033 |
|  | 3. Nationality of the Pharmacist (if known): | -2.938 | 221.834 | .000 | 1 | .989 | .053 |
|  | 1. What was the duration of the interaction?  (Start your mobile timer when the pharmacist begins the conversation, and stop it when the interaction ends.)  _______ minutes  Put numbers only (e.g., 0.5, 1, 1.5, 2, 2.5,3 ... etc.). | .991 | 1.461 | .459 | 1 | .498 | 2.693 |
|  | 2. Did the pharmacist ask about the pattern of sleep difficulty? | 7.417 | 7.103 | 1.091 | 1 | .296 | 1664.663 |
|  | 3. Did the pharmacist ask about the duration of the sleep problem? | 8.451 | 8.602 | .965 | 1 | .326 | 4679.876 |
|  | 4. Did the pharmacist ask if there were any triggers for the sleep problem (including medical, psychological, lifestyle and behavioral, environmental, and changes in usual routine)? | -1.527 | 6.299 | .059 | 1 | .808 | .217 |
|  | 5. Did the pharmacist ask about other symptoms accompanying your sleeplessness? | -8.263 | 12.199 | .459 | 1 | .498 | .000 |
|  | 6. Did the pharmacist ask about any actions you have already taken (if any)? | 32.538 | 2654.398 | .000 | 1 | .990 | 135251832236710.980 |
|  | 7. Did the pharmacist ask about the presence of any chronic health problems? | 19.873 | 5028.204 | .000 | 1 | .997 | 427283192.058 |
|  | 2. If a product was provided, provide the name and the strength of the product. | .579 | .388 | 2.231 | 1 | .135 | 1.784 |
|  | @3safetyadvicegivenbypharmacistx | -8.831 | 6.460 | 1.869 | 1 | .172 | .000 |
|  | 1. Did the pharmacist introduce himself/herself? | -18.789 | 1040.703 | .000 | 1 | .986 | .000 |
|  | 2. Did the pharmacist have a professional appearance (e.g., lab coat, name badge)? | -55.752 | 3409.235 | .000 | 1 | .987 | .000 |
|  | 3. Was your privacy ensured during the consultation? | 6.008 | 10.494 | .328 | 1 | .567 | 406.624 |
|  | 4. Did the pharmacist explain the reason for asking questions? | 22.012 | 5118.815 | .000 | 1 | .997 | 3628261361.367 |
|  | 5. Did the pharmacist avoid using inappropriate language (e.g., medical jargon or overly technical terms)? | 8.436 | 6.804 | 1.537 | 1 | .215 | 4608.491 |
|  | 6. Did the pharmacist ask if you needed any additional information or had any questions? | -26.644 | 1017.207 | .001 | 1 | .979 | .000 |
|  | 7. Did the pharmacist consider your preferences (e.g., medication choice, 29 form)? | -.993 | 2.804 | .125 | 1 | .723 | .370 |
|  | 8. Did the pharmacist check your understanding of the recommendations ? | 17.747 | 9.974 | 3.166 | 1 | .075 | 50998126.142 |
|  | 9. Did the pharmacist offer a way to follow up (e.g., provide a phone number or invite you to return to the pharmacy)? | 16.023 | 6251.726 | .000 | 1 | .998 | 9094134.973 |
|  | 10. Did the pharmacist tell you when you should visit a doctor? | 29.313 | 10750.669 | .000 | 1 | .998 | 5377592075752.742 |
|  | Constant | -160.711 | 33478.624 | .000 | 1 | .996 | .000 |

| a. Variable(s) entered on step 1: 1. The region in which the pharmacy is located , 2. The pharmacy is located in, 3. Type of pharmacy, 4. Time of Visit, 5. Day of Visit, 6. Dispensary Load (Number of customers at the time of visit), 1. Estimated Age of the Pharmacist:  , 2. Gender of the Pharmacist  , 3. Nationality of the Pharmacist (if known):, 1. What was the duration of the interaction?  (Start your mobile timer when the pharmacist begins the conversation, and stop it when the interaction ends.)  _______ minutes  Put numbers only (e.g., 0.5, 1, 1.5, 2, 2.5,3 ... etc.)., 2. Did the pharmacist ask about the pattern of sleep difficulty?  , 3. Did the pharmacist ask about the duration of the sleep problem?, 4. Did the pharmacist ask if there were any triggers for the sleep problem (including medical, psychological, lifestyle and behavioral, environmental, and changes in usual routine)?, 5. Did the pharmacist ask about other symptoms accompanying your sleeplessness?, 6. Did the pharmacist ask about any actions you have already taken (if any)?, 7. Did the pharmacist ask about the presence of any chronic health problems?, 2. If a product was provided, provide the name and the strength of the product. , @3safetyadvicegivenbypharmacistx, 1. Did the pharmacist introduce himself/herself?, 2. Did the pharmacist have a professional appearance (e.g., lab coat, name badge)?, 3. Was your privacy ensured during the consultation?, 4. Did the pharmacist explain the reason for asking questions?, 5. Did the pharmacist avoid using inappropriate language (e.g., medical jargon or overly technical terms)?, 6. Did the pharmacist ask if you needed any additional information or had any questions?, 7. Did the pharmacist consider your preferences (e.g., medication choice, 29 form)?, 8. Did the pharmacist check your understanding of the recommendations ?, 9. Did the pharmacist offer a way to follow up (e.g., provide a phone number or invite you to return to the pharmacy)? , 10. Did the pharmacist tell you when you should visit a doctor?. |
| --- |

| **Casewise List^b^** | | | | | | | |
| --- | --- | --- | --- | --- | --- | --- | --- |
| Case | Selected Status^a^ | Observed | Predicted | Predicted Group | Temporary Variable | | |
|  |  | Non drug recommendation |  |  | Resid | ZResid | SResid |
| 135 | S | N | .358 | N | -.358 | -.747 | -4.189 |
| 139 | S | Y** | .420 | N | .580 | 1.174 | 2.453 |
| 201 | S | Y** | .310 | N | .690 | 1.493 | 3.246 |
| 217 | S | Y** | .145 | N | .855 | 2.433 | 2.780 |
| 218 | S | N** | .725 | Y | -.725 | -1.625 | -3.292 |

| a. S = Selected, U = Unselected cases, and ** = Misclassified cases. |
| --- |
| b. Cases with studentized residuals greater than 2.000 are listed. |
