# Supplementary material for: Evaluating community pharmacists’ practices in managing insomnia in Saudi Arabia: a cross-sectional study using simulated patient visits
Source: Front Public Health. 2026 Jun 16;14:1792148. doi: 10.3389/fpubh.2026.1792148 (PMC13326896; doi:10.3389/fpubh.2026.1792148)
Supplement: Supplementary file 4 [file Table_2.DOCX]

**Table 1: The pharmacist demographics and pharmacy visit related characteristics**

| **Variable** | **Sub variable** | **Frequency** | **Percentage** |
| --- | --- | --- | --- |
| The region in which the pharmacy is located | Makkah | 92 | 36.7 |
|  | Jazan | 107 | 42.6 |
|  | Al Baha | 52 | 20.7 |
| Location of pharmacy | Urban Area | 109 | 43.4 |
|  | Suburban Area | 124 | 49.4 |
|  | Rural | 18 | 7.2 |
| Type of pharmacy | National Chain | 120 | 47.8 |
|  | Local Chain | 65 | 25.9 |
|  | Independent | 66 | 26.3 |
| Time of visit | Morning shift (8:00 AM - 3:00 PM) | 58 | 23.1 |
|  | Evening shift (3:00 PM to 11:00 PM) | 155 | 61.8 |
|  | Night shift (11:00 PM to 7:00 AM) | 38 | 15.1 |
| Day of visit | Weekday | 154 | 61.4 |
|  | Weekend | 97 | 38.6 |
| Dispensary load (Number of patients at the time of visit) | Quiet (Zero customers) | 139 | 55.4 |
|  | Low (1 or 2 customers) | 82 | 32.7 |
|  | Moderate (3 to 5 customers) | 17 | 6.8 |
|  | Busy (More than 5 customers) | 13 | 5.2 |
| Estimated age of pharmacist | Less than 30 years | 85 | 33.9 |
|  | 30 - 40 Years | 121 | 48.2 |
|  | More than 40 years | 45 | 17.9 |
| Gender of pharmacist | Male | 235 | 93.6 |
|  | Female | 16 | 6.4 |
| Nationality of pharmacist | Saudi | 44 | 17.5 |
|  | Non-Saudi | 207 | 82.5 |

**Table 2: The descriptive statistics of pharmacist consultation process**

| **Variable** | **Sub variable** | **Frequency** | **Percentage** |
| --- | --- | --- | --- |
| Duration of pharmacist interaction | .25 minutes | 2 | .8 |
|  | 0.5 minutes | 21 | 8.4 |
|  | .75 minutes | 1 | .4 |
|  | 1 minute | 63 | 25.1 |
|  | 1.5 minutes | 73 | 29.1 |
|  | 2 minutes | 54 | 21.5 |
|  | 2.5 minutes | 23 | 9.2 |
|  | 3 minutes | 6 | 2.4 |
|  | 3.5 minutes | 4 | 1.6 |
|  | 4 minutes | 1 | .4 |
|  | 5 minutes | 1 | .4 |
|  | 9 minutes | 1 | .4 |
|  | 22 minutes | 1 | .4 |
| Did the pharmacist ask about the pattern of sleep difficulty? | Yes | 139 | 55.4 |
|  | No | 112 | 44.6 |
| Did the pharmacist ask about the duration of the sleep problem? | Yes | 113 | 45.0 |
|  | No | 138 | 55.0 |
| Did the pharmacist ask if there were any triggers for the sleep problem (including medical, psychological, lifestyle and behavioural, environmental, and changes in usual routine)? | Yes | 128 | 51.0 |
|  | No | 123 | 49.0 |
| Did the pharmacist ask about other symptoms accompanying your sleeplessness? | Yes | 44 | 17.5 |
|  | No | 207 | 82.5 |
| Did the pharmacist ask about any actions you have already taken? | Yes | 71 | 28.3 |
|  | No | 180 | 71.7 |
| Did the pharmacist ask about the presence of any chronic health problems? | Yes | 46 | 18.3 |
|  | No | 205 | 81.7 |

**Table 3: The descriptive statistics of pharmacist action process**

| **Variable** | **Sub variable** | **Frequency** | **Percentage** |
| --- | --- | --- | --- |
| What action did the pharmacist take? | Supplied an OTC product | 227 | 90.4 |
|  | Supplied an OTC product and provided non-pharmacological advice | 17 | 6.8 |
|  | Provided non-pharmacological advice | 7 | 2.8 |
| If a product was provided, provide the name and the strength of the product. | None | 6 | 2.4 |
|  | Melatonin (1mg to 5mg) | 162 | 64.5 |
|  | MEL + PCT (500 mg)+DPH (25 mg) | 4 | 1.6 |
|  | MEL + Ashwagandha (300 mg) | 3 | 1.2 |
|  | Valerian root (120 mg)+Melissa leaf (80 mg) | 12 | 4.8 |
|  | Magnesium pills | 4 | 1.6 |
|  | Strep wills night water or syrup | 3 | 1.2 |
|  | Chlorpheniramine | 1 | .4 |
|  | PCT + DPH | 44 | 17.5 |
|  | Ashwagandha | 2 | .8 |
|  | MEL + Melissa | 2 | .8 |
|  | Primrose oil | 1 | .4 |
|  | MEL + Valerian | 1 | .4 |
|  | DPH + Dextromethorphan | 1 | .4 |
|  | Dream water | 3 | 1.2 |
|  | Valerian | 1 | .4 |
|  | MEL + St.john's wart root | 1 | .4 |
| What information did the pharmacist provide about the product? | Generic name | 95 | 42.2% |
|  | Administration times (when and how often to take it) | 92 | 40.9% |
|  | Brand name | 81 | 36.0% |
|  | Dosage | 47 | 20.9% |
|  | Dosage form (e.g., tablet, capsule, syrup) | 47 | 20.9% |
|  | Side effects and warnings | 11 | 4.9% |
|  | Time needed for the medication to take effect | 3 | 1.3% |
|  | Maximum recommended duration of use | 2 | 0.9% |
| If non-pharmacological advice was given, which of the following topics were addressed? | Reducing caffeine intake, especially in the evening | 78 | 32.4% |
|  | Limiting screen time before bed | 25 | 10.4% |
|  | Establishing a regular sleep schedule | 20 | 8.3% |
|  | Creating a comfortable sleep environment (dark, quiet, cool) | 15 | 6.2% |
|  | Going to bed only when sleepy | 12 | 5.0% |
|  | Avoiding heavy meals before bedtime | 10 | 4.2% |
|  | Avoiding naps late in the day | 9 | 3.7% |
|  | Using relaxation techniques (...) | 6 | 2.5% |
|  | None/Empty/N/A/Non | 78 | 32.4% |
|  | Other (Exercise, Marriage) | 2 | 0.8% |

MEL = Melatonin

PCT = Paracetamol

DPH = Diphenhydramine

**Table 4: The descriptive statistics of pharmacist’s professionalism, communication and interaction**

| **Variable** | **Sub variable** | **Frequency** | **Percentage** |
| --- | --- | --- | --- |
| Did the pharmacist introduce himself/herself? | Yes | 38 | 15.1 |
|  | No | 213 | 84.9 |
| Did the pharmacist have a professional appearance (e.g., lab coat, name badge)? | Yes | 215 | 85.7 |
|  | No | 36 | 14.3 |
| Was your privacy ensured during the consultation? | Yes | 120 | 47.8 |
|  | No | 131 | 52.2 |
| Did the pharmacist explain the reason for asking questions? | Yes | 51 | 20.3 |
|  | No | 200 | 79.7 |
| Did the pharmacist avoid using inappropriate language (e.g., medical jargon or overly technical terms)? | Yes | 240 | 95.6 |
|  | No | 11 | 4.4 |
| Did the pharmacist ask if you needed any additional information or had any questions? | Yes | 39 | 15.5 |
|  | No | 212 | 84.5 |
| Did the pharmacist consider your preferences (e.g., medication choice, 29 form)? | Yes | 48 | 19.1 |
|  | No | 203 | 80.9 |
| Did the pharmacist check your understanding of the recommendations? | Yes | 98 | 39.0 |
|  | No | 153 | 61.0 |
| Did the pharmacist offer a way to follow up (e.g., provide a phone number or invite you to return to the pharmacy)? | Yes | 12 | 4.8 |
|  | No | 239 | 95.2 |
| Did the pharmacist tell you when you should visit a doctor? | Yes | 15 | 6.0 |
|  | No | 236 | 94.0 |

**Table 5: Chi-square associations between pharmacy framework, pharmacist demographics and consultation quality indicators**

| **Main variable** | **Associate variable** | **χ² - value** | **df** | **p-value** | **Significance** |
| --- | --- | --- | --- | --- | --- |
| The region in which the pharmacy is located: Jazan, Makkah, Al Baha | Did the pharmacist ask about the pattern of sleep difficulty? | 69.899 | 2 | .000 | Extremely significant |
|  | Did the pharmacist ask about the duration of the sleep problem? | 85.228 | 2 | .000 | Extremely significant |
|  | Did the pharmacist ask if there were any triggers for the sleep problem (including medical, psychological, lifestyle and behavioural, environmental, and changes in usual routine)? | 101.994 | 2 | .000 | Extremely significant |
|  | Did the pharmacist ask about other symptoms accompanying your sleeplessness? | 55.751 | 2 | .000 | Extremely significant |
|  | Did the pharmacist ask about any actions you have already taken (if any)? | 24.134 | 2 | .000 | Extremely significant |
|  | Did the pharmacist ask about the presence of any chronic health problems? | 41.390 | 2 | .000 | Extremely significant |
|  | What action did the pharmacist take? (Select all that apply.) | 10.672 | 4 | .031 | Significant |
|  | If a product was provided, provide the name and the strength of the product. | 97.273 | 32 | .000 | Extremely significant |
|  | Did the pharmacist introduce himself/herself? | 54.958 | 2 | .000 | Extremely significant |
|  | Did the pharmacist have a professional appearance (e.g., lab coat, name badge)? | .069 | 2 | .966 | Insignificant |
|  | Was your privacy ensured during the consultation? | 111.015 | 2 | .000 | Extremely significant |
|  | Did the pharmacist explain the reason for asking questions? | 80.374 | 2 | .000 | Extremely significant |
|  | Did the pharmacist avoid using inappropriate language (e.g., medical jargon or overly technical terms)? | 2.452 | 2 | .293 | Insignificant |
|  | Did the pharmacist ask if you needed any additional information or had any questions? | 43.407 | 2 | .000 | Extremely significant |
|  | Did the pharmacist consider your preferences (e.g., medication choice, Dosage form)? | 15.936 | 2 | .000 | Extremely significant |
|  | Did the pharmacist check your understanding of the recommendations? | 128.548 | 2 | .000 | Extremely significant |
|  | Did the pharmacist offer a way to follow up (e.g., provide a phone number or invite you to return to the pharmacy)? | 16.960 | 2 | .000 | Extremely significant |
|  | Did the pharmacist tell you when you should visit a doctor? | 12.691 | 2 | .002 | Highly significant |
| Pharmacy location: Urban, Suburban and Rural settings | Asked about pattern of sleep difficulty | 9.234 | 2 | 0.01 | Highly significant |
|  | Asked about duration of sleep problem | 4.651 | 2 | 0.098 | Insignificant |
|  | Asked about triggers for sleep problem | 11.61 | 2 | 0.003 | Highly significant |
|  | Asked about other symptoms | 4.049 | 2 | 0.132 | Insignificant |
|  | Asked about actions already taken | 1.34 | 2 | 0.512 | Insignificant |
|  | Asked about chronic health problems | 0.211 | 2 | 0.9 | Insignificant |
|  | What action did the pharmacist take | 2.207 | 4 | 0.698 | Insignificant |
|  | Product name and strength provided | 30.657 | 32 | 0.534 | Insignificant |
|  | Did the pharmacist introduce himself/herself | 0.961 | 2 | 0.618 | Insignificant |
|  | Had professional appearance | 1.866 | 2 | 0.393 | Insignificant |
|  | Was your privacy ensured | 7.382 | 2 | 0.025 | Significant |
|  | Explained reason for asking questions | 6.625 | 2 | 0.036 | Significant |
|  | Avoided inappropriate language | 1.189 | 2 | 0.552 | Insignificant |
|  | Asked if you needed additional information | 10.19 | 2 | 0.006 | Highly significant |
|  | Considered your preferences | 0.14 | 2 | 0.933 | Insignificant |
|  | Checked your understanding of recommendations | 9.011 | 2 | 0.011 | Significant |
|  | Offered a way to follow up | 7.23 | 2 | 0.027 | Significant |
|  | Told you when to visit a doctor | 0.652 | 2 | 0.722 | Insignificant |

**Table 6: Chi-square associations between type of pharmacy, time and day of consultation and quality indicators**

| Type of pharmacy | Asked about pattern of sleep difficulty | 9.929 | 2 | 0.007 | Highly significant |
| --- | --- | --- | --- | --- | --- |
|  | Asked about duration of sleep problem | 6.554 | 2 | 0.038 | Significant |
|  | Asked about triggers for sleep problem | 14.648 | 2 | 0.001 | Extremely significant |
|  | Asked about other symptoms | 6.274 | 2 | 0.043 | Significant |
|  | Asked about actions already taken | 10.145 | 2 | 0.006 | Highly significant |
|  | Asked about chronic health problems | 3.188 | 2 | 0.203 | Insignificant |
|  | What action did the pharmacist take | 2.014 | 4 | 0.733 | Insignificant |
|  | Product name and strength provided | 52.205 | 32 | 0.014 | Significant |
|  | Did the pharmacist introduce himself/herself | 5.056 | 2 | 0.08 | Insignificant |
|  | Had professional appearance | 8.802 | 2 | 0.012 | Significant |
|  | Was your privacy ensured | 4.783 | 2 | 0.092 | Insignificant |
|  | Explained reason for asking questions | 8.926 | 2 | 0.012 | Significant |
|  | Avoided inappropriate language | 0.767 | 2 | 0.681 | Insignificant |
|  | Asked if you needed additional information | 5.171 | 2 | 0.075 | Insignificant |
|  | Considered your preferences | 2.55 | 2 | 0.279 | Insignificant |
|  | Checked your understanding of recommendations | 18.717 | 2 | .000 | Extremely significant |
|  | Offered a way to follow up | 3.66 | 2 | 0.16 | Insignificant |
|  | Told you when to visit a doctor | 9.87 | 2 | 0.007 | Highly significant |
| Time of Visit | Asked about pattern of sleep difficulty | 19.517 | 2 | .000 | Extremely significant |
|  | Asked about duration of sleep problem | 21.049 | 2 | .000 | Extremely significant |
|  | Asked about triggers for sleep problem | 27.615 | 2 | .000 | Extremely significant |
|  | Asked about other symptoms | 19.2 | 2 | .000 | Extremely significant |
|  | Asked about actions already taken | 1.976 | 2 | 0.372 | Insignificant |
|  | Asked about chronic health problems | 14.729 | 2 | 0.001 | Extremely significant |
|  | What action did the pharmacist take | 4.361 | 4 | 0.359 | Insignificant |
|  | Product name and strength provided | 56.737 | 32 | 0.005 | Highly significant |
|  | Did the pharmacist introduce himself/herself | 24.207 | 2 | .000 | Extremely significant |
|  | Had professional appearance | 1.515 | 2 | 0.469 | Insignificant |
|  | Was your privacy ensured | 27.099 | 2 | .000 | Extremely significant |
|  | Explained reason for asking questions | 32.379 | 2 | .000 | Extremely significant |
|  | Avoided inappropriate language | 7.157 | 2 | 0.028 | Significant |
|  | Asked if you needed additional information | 14.671 | 2 | 0.001 | Extremely significant |
|  | Considered your preferences | 1.004 | 2 | 0.605 | Insignificant |
|  | Checked your understanding of recommendations | 30.604 | 2 | .000 | Extremely significant |
|  | Offered a way to follow up | 3.598 | 2 | 0.165 | Insignificant |
|  | Told you when to visit a doctor | 15.7 | 2 | .000 | Extremely significant |
| Day of Visit | Asked about pattern of sleep difficulty | 0.502 | 1 | 0.479 | Insignificant |
|  | Asked about duration of sleep problem | 0.914 | 1 | 0.339 | Insignificant |
|  | Asked about triggers for sleep problem | 7.365 | 1 | 0.007 | Highly significant |
|  | Asked about other symptoms | 0 | 1 | 0.999 | Insignificant |
|  | Asked about actions already taken | 0.544 | 1 | 0.461 | Insignificant |
|  | Asked about chronic health problems | 2.562 | 1 | 0.109 | Insignificant |
|  | What action did the pharmacist take | 0.615 | 2 | 0.735 | Insignificant |
|  | Product name and strength provided | 21.02 | 16 | 0.178 | Insignificant |
|  | Did the pharmacist introduce himself/herself | 4.227 | 1 | 0.04 | Significant |
|  | Had professional appearance | 0.001 | 1 | 0.974 | Insignificant |
|  | Was your privacy ensured | 0.009 | 1 | 0.923 | Insignificant |
|  | Explained reason for asking questions | 2.302 | 1 | 0.129 | Insignificant |
|  | Avoided inappropriate language | 0.628 | 1 | 0.428 | Insignificant |
|  | Asked if you needed additional information | 0.11 | 1 | 0.74 | Insignificant |
|  | Considered your preferences | 4.661 | 1 | 0.031 | Significant |
|  | Checked your understanding of recommendations | 2.435 | 1 | 0.119 | Insignificant |
|  | Offered a way to follow up | 0.15 | 1 | 0.699 | Insignificant |
|  | Told you when to visit a doctor | 4.311 | 1 | 0.038 | Significant |
| Dispensary Load | Asked about pattern of sleep difficulty | 3.943 | 3 | 0.268 | Insignificant |
|  | Asked about duration of sleep problem | 3.471 | 3 | 0.324 | Insignificant |
|  | Asked about triggers for sleep problem | 0.736 | 3 | 0.865 | Insignificant |
|  | Asked about other symptoms | 2.911 | 3 | 0.406 | Insignificant |
|  | Asked about actions already taken | 0.904 | 3 | 0.825 | Insignificant |
|  | Asked about chronic health problems | 5.422 | 3 | 0.143 | Insignificant |
|  | What action did the pharmacist take | 5.051 | 6 | 0.537 | Insignificant |
|  | Product name and strength provided | 72.244 | 48 | 0.013 | Significant |
|  | Did the pharmacist introduce himself/herself | 2.22 | 3 | 0.528 | Insignificant |
|  | Had professional appearance | 10.956 | 3 | 0.012 | Significant |
|  | Was your privacy ensured | 41.37 | 3 | .000 | Extremely significant |
|  | Explained reason for asking questions | 4.525 | 3 | 0.21 | Insignificant |
|  | Avoided inappropriate language | 0.535 | 3 | 0.911 | Insignificant |
|  | Asked if you needed additional information | 2.555 | 3 | 0.465 | Insignificant |
|  | Considered your preferences | 4.097 | 3 | 0.251 | Insignificant |
|  | Checked your understanding of recommendations | 1.293 | 3 | 0.731 | Insignificant |
|  | Offered a way to follow up | 1.215 | 3 | 0.749 | Insignificant |
|  | Told you when to visit a doctor | 2.823 | 3 | 0.42 | Insignificant |
| Estimated Age of Pharmacist | Asked about pattern of sleep difficulty | 0.312 | 2 | 0.855 | Insignificant |
|  | Asked about duration of sleep problem | 2.006 | 2 | 0.367 | Insignificant |
|  | Asked about triggers for sleep problem | 2.922 | 2 | 0.232 | Insignificant |
|  | Asked about other symptoms | 3.254 | 2 | 0.197 | Insignificant |
|  | Asked about actions already taken | 1.437 | 2 | 0.487 | Insignificant |
|  | Asked about chronic health problems | 0.073 | 2 | 0.964 | Insignificant |
|  | What action did the pharmacist take | 3.487 | 4 | 0.48 | Insignificant |
|  | Product name and strength provided | 36.472 | 32 | 0.268 | Insignificant |
|  | Did the pharmacist introduce himself/herself | 1.179 | 2 | 0.555 | Insignificant |
|  | Had professional appearance | 6.346 | 2 | 0.042 | Significant |
|  | Was your privacy ensured | 0.569 | 2 | 0.753 | Insignificant |
|  | Explained reason for asking questions | 2.703 | 2 | 0.259 | Insignificant |
|  | Avoided inappropriate language | 3.213 | 2 | 0.201 | Insignificant |
|  | Asked if you needed additional information | 0.216 | 2 | 0.898 | Insignificant |
|  | Considered your preferences | 2.758 | 2 | 0.252 | Insignificant |
|  | Checked your understanding of recommendations | 9.243 | 2 | 0.01 | Highly significant |
|  | Offered a way to follow up | 0.652 | 2 | 0.722 | Insignificant |
|  | Told you when to visit a doctor | 4.185 | 2 | 0.123 | Insignificant |
| Gender of Pharmacist | Asked about pattern of sleep difficulty | 4.629 | 1 | 0.031 | Significant |
|  | Asked about duration of sleep problem | 2.11 | 1 | 0.146 | Insignificant |
|  | Asked about triggers for sleep problem | 6.259 | 1 | 0.012 | Significant |
|  | Asked about other symptoms | 4.714 | 1 | 0.03 | Significant |
|  | Asked about actions already taken | 2.1 | 1 | 0.147 | Insignificant |
|  | Asked about chronic health problems | 1.907 | 1 | 0.167 | Insignificant |
|  | What action did the pharmacist take | 1.321 | 2 | 0.517 | Insignificant |
|  | Product name and strength provided | 7.706 | 16 | 0.957 | Insignificant |
|  | Did the pharmacist introduce himself/herself | 38.231 | 1 | .000 | Extremely significant |
|  | Had professional appearance | 0.047 | 1 | 0.828 | Insignificant |
|  | Was your privacy ensured | 1.478 | 1 | 0.224 | Insignificant |
|  | Explained reason for asking questions | 0.231 | 1 | 0.631 | Insignificant |
|  | Avoided inappropriate language | 2.687 | 1 | 0.101 | Insignificant |
|  | Asked if you needed additional information | 3.215 | 1 | 0.073 | Insignificant |
|  | Considered your preferences | 1.831 | 1 | 0.176 | Insignificant |
|  | Checked your understanding of recommendations | 3.951 | 1 | 0.047 | Significant |
|  | Offered a way to follow up | 0.081 | 1 | 0.776 | Insignificant |
|  | Told you when to visit a doctor | 0.002 | 1 | 0.962 | Insignificant |
| Nationality of Pharmacist | Asked about pattern of sleep difficulty | 3.539 | 1 | 0.06 | Insignificant |
|  | Asked about duration of sleep problem | 5.758 | 1 | 0.016 | Significant |
|  | Asked about triggers for sleep problem | 2.295 | 1 | 0.13 | Insignificant |
|  | Asked about other symptoms | 0.316 | 1 | 0.574 | Insignificant |
|  | Asked about actions already taken | 2.686 | 1 | 0.101 | Insignificant |
|  | Asked about chronic health problems | 11.596 | 1 | 0.001 | Extremely significant |
|  | What action did the pharmacist take | 3.999 | 2 | 0.135 | Insignificant |
|  | Product name and strength provided | 14.299 | 16 | 0.576 | Insignificant |
|  | Did the pharmacist introduce himself/herself | 18.707 | 1 | .000 | Extremely significant |
|  | Had professional appearance | 2.459 | 1 | 0.117 | Insignificant |
|  | Was your privacy ensured | 1.736 | 1 | 0.188 | Insignificant |
|  | Explained reason for asking questions | 0.191 | 1 | 0.662 | Insignificant |
|  | Avoided inappropriate language | 0.755 | 1 | 0.385 | Insignificant |
|  | Asked if you needed additional information | 2.101 | 1 | 0.147 | Insignificant |
|  | Considered your preferences | 1.191 | 1 | 0.275 | Insignificant |
|  | Checked your understanding of recommendations | 0.921 | 1 | 0.337 | Insignificant |
|  | Offered a way to follow up | 0.486 | 1 | 0.486 | Insignificant |
|  | Told you when to visit a doctor | 0.921 | 1 | 0.337 | Insignificant |
| Duration of interaction | Asked about pattern of sleep difficulty | 60.244 | 12 | .000 | Extremely significant |
|  | Asked about duration of sleep problem | 42.918 | 12 | .000 | Extremely significant |
|  | Asked about triggers for sleep problem | 46.654 | 12 | .000 | Extremely significant |
|  | Asked about other symptoms | 35.835 | 12 | .000 | Extremely significant |
|  | Asked about actions already taken | 15.167 | 12 | 0.232 | Insignificant |
|  | Asked about chronic health problems | 23.067 | 12 | 0.027 | Significant |
|  | What action did the pharmacist take | 94.004 | 24 | .000 | Extremely significant |
|  | Product name and strength provided | 778.701 | 192 | .000 | Extremely significant |
|  | Did the pharmacist introduce himself/herself | 27.769 | 12 | 0.006 | Highly significant |
|  | Had professional appearance | 5.205 | 12 | 0.951 | Insignificant |
|  | Was your privacy ensured | 18.375 | 12 | 0.105 | Insignificant |
|  | Explained reason for asking questions | 16.355 | 12 | 0.176 | Insignificant |
|  | Avoided inappropriate language | 25.024 | 12 | 0.015 | Significant |
|  | Asked if you needed additional information | 31.256 | 12 | 0.002 | Highly significant |
|  | Considered your preferences | 26.607 | 12 | 0.009 | Highly significant |
|  | Checked your understanding of recommendations | 39.072 | 12 | .000 | Extremely significant |
|  | Offered a way to follow up | 6.551 | 12 | 0.886 | Insignificant |
|  | Told you when to visit a doctor | 19.508 | 12 | 0.077 | Insignificant |

**Table 6: Correlation analysis between pharmacy framework, pharmacist demographics and consultation quality indicators**

| **Variable 1** | **Variable 2** | **R-value** | **p-value** | **Interpretation** |
| --- | --- | --- | --- | --- |
| The region in which the pharmacy is located | Time of Visit | -0.212 | 0.001 | Weak negative correlation |
|  | What was the duration of the interaction? | -0.208 | 0.001 | Weak negative correlation |
|  | Did the pharmacist ask about any actions you have already taken (if any)? | -0.135 | 0.032 | Weak negative correlation |
|  | If a product was provided, provide the name and the strength of the product. | 0.219 | 0 | Weak positive correlation |
|  | Was your privacy ensured during the consultation? | -0.605 | 0 | Moderate negative correlation |
|  | Did the pharmacist ask if you needed any additional information or had any questions? | -0.152 | 0.016 | Weak negative correlation |
|  | Did the pharmacist consider your preferences (e.g., medication choice, form)? | 0.224 | 0 | Weak positive correlation |
|  | Did the pharmacist check your understanding of the recommendations? | -0.183 | 0.004 | Weak negative correlation |
| The pharmacy is located in | Type of pharmacy | 0.144 | 0.023 | Weak positive correlation |
|  | Day of Visit | -0.345 | 0 | Moderate negative correlation |
|  | Estimated Age of the Pharmacist | 0.125 | 0.048 | Weak positive correlation |
|  | Gender of the Pharmacist | -0.139 | 0.028 | Weak negative correlation |
|  | Did the pharmacist check your understanding of the recommendations? | -0.127 | 0.044 | Weak negative correlation |
| Type of pharmacy | Dispensary Load (Number of customers at the time of visit) | -0.224 | 0 | Weak negative correlation |
|  | Estimated Age of the Pharmacist | 0.261 | 0 | Weak positive correlation |
|  | Gender of the Pharmacist | -0.187 | 0.003 | Weak negative correlation |
|  | Nationality of the Pharmacist (if known) | 0.271 | 0 | Weak positive correlation |
|  | Did the pharmacist ask about the duration of the sleep problem? | -0.137 | 0.029 | Weak negative correlation |
|  | Did the pharmacist ask if there were any triggers for the sleep problem? | -0.168 | 0.008 | Weak negative correlation |
|  | Did the pharmacist ask about any actions you have already taken (if any)? | -0.151 | 0.016 | Weak negative correlation |
|  | If a product was provided, provide the name and the strength of the product. | 0.153 | 0.015 | Weak positive correlation |
|  | Did the pharmacist have a professional appearance (e.g., lab coat, name badge)? | 0.174 | 0.006 | Weak positive correlation |
|  | Did the pharmacist explain the reason for asking questions? | -0.154 | 0.015 | Weak negative correlation |
|  | Did the pharmacist tell you when you should visit a doctor? | -0.146 | 0.021 | Weak negative correlation |
| Time of Visit | Day of Visit | 0.37 | 0 | Moderate positive correlation |
|  | What was the duration of the interaction? | 0.167 | 0.008 | Weak positive correlation |
|  | Was your privacy ensured during the consultation? | 0.149 | 0.018 | Weak positive correlation |
|  | Did the pharmacist avoid using inappropriate language? | -0.163 | 0.01 | Weak negative correlation |
| Day of Visit | Nationality of the Pharmacist (if known) | 0.151 | 0.017 | Weak positive correlation |
|  | Did the pharmacist ask if there were any triggers for the sleep problem? | 0.171 | 0.007 | Weak positive correlation |
|  | Did the pharmacist introduce himself/herself? | 0.13 | 0.04 | Weak positive correlation |
|  | Did the pharmacist consider your preferences? | 0.136 | 0.031 | Weak positive correlation |
|  | Did the pharmacist tell you when you should visit a doctor? | 0.131 | 0.038 | Weak positive correlation |
| Estimated Age of the Pharmacist | Gender of the Pharmacist | -0.173 | 0.006 | Weak negative correlation |
|  | Nationality of the Pharmacist (if known) | 0.298 | 0 | Weak positive correlation |
|  | Did the pharmacist have a professional appearance? | 0.158 | 0.012 | Weak positive correlation |
|  | Did the pharmacist check your understanding of the recommendations? | -0.182 | 0.004 | Weak negative correlation |
|  | Did the pharmacist tell you when you should visit a doctor? | -0.129 | 0.041 | Weak negative correlation |
| Gender of the Pharmacist | Nationality of the Pharmacist (if known) | -0.437 | 0 | Moderate negative correlation |
|  | What was the duration of the interaction? | 0.213 | 0.001 | Weak positive correlation |
|  | Did the pharmacist ask about the pattern of sleep difficulty? | -0.136 | 0.031 | Weak negative correlation |
|  | Did the pharmacist ask if there were any triggers for the sleep problem? | -0.158 | 0.012 | Weak negative correlation |
|  | Did the pharmacist ask about other symptoms accompanying your sleeplessness? | -0.137 | 0.03 | Weak negative correlation |
|  | Did the pharmacist introduce himself/herself? | -0.39 | 0 | Weak negative correlation |
|  | Did the pharmacist check your understanding of the recommendations? | -0.125 | 0.047 | Weak negative correlation |
| Nationality of the Pharmacist (if known) | Did the pharmacist ask about the duration of the sleep problem? | 0.151 | 0.016 | Weak positive correlation |
|  | Did the pharmacist ask about the presence of any chronic health problems? | 0.215 | 0.001 | Weak positive correlation |
|  | Did the pharmacist introduce himself/herself? | 0.273 | 0 | Weak positive correlation |
| What was the duration of the interaction? | Did the pharmacist ask about the pattern of sleep difficulty? | -0.25 | 0 | Weak negative correlation |
|  | Did the pharmacist ask about the duration of the sleep problem? | -0.204 | 0.001 | Weak negative correlation |
|  | Did the pharmacist ask if there were any triggers for the sleep problem? | -0.211 | 0.001 | Weak negative correlation |
|  | Did the pharmacist ask about other symptoms accompanying your sleeplessness? | -0.219 | 0 | Weak negative correlation |
|  | Did the pharmacist explain the reason for asking questions? | -0.124 | 0.049 | Weak negative correlation |
|  | Did the pharmacist ask if you needed any additional information or had any questions? | -0.207 | 0.001 | Weak negative correlation |
|  | Did the pharmacist check your understanding of the recommendations? | -0.182 | 0.004 | Weak negative correlation |
| Did the pharmacist ask about the pattern of sleep difficulty? | Did the pharmacist ask about the duration of the sleep problem? | 0.506 | 0 | Moderate positive correlation |
|  | Did the pharmacist ask if there were any triggers for the sleep problem? | 0.531 | 0 | Moderate positive correlation |
|  | Did the pharmacist ask about other symptoms accompanying your sleeplessness? | 0.266 | 0 | Weak positive correlation |
|  | Did the pharmacist ask about any actions you have already taken (if any)? | 0.172 | 0.006 | Weak positive correlation |
|  | Did the pharmacist ask about the presence of any chronic health problems? | 0.259 | 0 | Weak positive correlation |
|  | Did the pharmacist introduce himself/herself? | 0.29 | 0 | Weak positive correlation |
|  | Was your privacy ensured during the consultation? | 0.201 | 0.001 | Weak positive correlation |
|  | Did the pharmacist explain the reason for asking questions? | 0.294 | 0 | Weak positive correlation |
|  | Did the pharmacist ask if you needed any additional information or had any questions? | 0.252 | 0 | Weak positive correlation |
|  | Did the pharmacist check your understanding of the recommendations? | 0.505 | 0 | Moderate positive correlation |
|  | Did the pharmacist offer a way to follow up (e.g., provide a phone number or invite you to return to the pharmacy)? | 0.201 | 0.001 | Weak positive correlation |
|  | Did the pharmacist tell you when you should visit a doctor? | 0.192 | 0.002 | Weak positive correlation |
| Did the pharmacist ask about the duration of the sleep problem? | Did the pharmacist ask if there were any triggers for the sleep problem? | 0.471 | 0 | Moderate positive correlation |
|  | Did the pharmacist ask about other symptoms accompanying your sleeplessness? | 0.278 | 0 | Weak positive correlation |
|  | Did the pharmacist ask about any actions you have already taken (if any)? | 0.25 | 0 | Weak positive correlation |
|  | Did the pharmacist ask about the presence of any chronic health problems? | 0.358 | 0 | Weak positive correlation |
|  | Did the pharmacist introduce himself/herself? | 0.31 | 0 | Weak positive correlation |
|  | Was your privacy ensured during the consultation? | 0.272 | 0 | Weak positive correlation |
|  | Did the pharmacist explain the reason for asking questions? | 0.399 | 0 | Weak positive correlation |
|  | Did the pharmacist ask if you needed any additional information or had any questions? | 0.319 | 0 | Weak positive correlation |
|  | Did the pharmacist consider your preferences? | 0.13 | 0.039 | Weak positive correlation |
|  | Did the pharmacist check your understanding of the recommendations? | 0.507 | 0 | Moderate positive correlation |
|  | Did the pharmacist offer a way to follow up? | 0.21 | 0.001 | Weak positive correlation |
|  | Did the pharmacist tell you when you should visit a doctor? | 0.245 | 0 | Weak positive correlation |
| Did the pharmacist ask if there were any triggers for the sleep problem? | Did the pharmacist ask about other symptoms accompanying your sleeplessness? | 0.368 | 0 | Weak positive correlation |
|  | Did the pharmacist ask about any actions you have already taken (if any)? | 0.244 | 0 | Weak positive correlation |
|  | Did the pharmacist ask about the presence of any chronic health problems? | 0.32 | 0 | Weak positive correlation |
|  | Did the pharmacist introduce himself/herself? | 0.347 | 0 | Weak positive correlation |
|  | Was your privacy ensured during the consultation? | 0.268 | 0 | Weak positive correlation |
|  | Did the pharmacist explain the reason for asking questions? | 0.396 | 0 | Weak positive correlation |
|  | Did the pharmacist ask if you needed any additional information or had any questions? | 0.354 | 0 | Weak positive correlation |
|  | Did the pharmacist check your understanding of the recommendations? | 0.539 | 0 | Moderate positive correlation |
|  | Did the pharmacist offer a way to follow up? | 0.182 | 0.004 | Weak positive correlation |
|  | Did the pharmacist tell you when you should visit a doctor? | 0.146 | 0.02 | Weak positive correlation |
| Did the pharmacist ask about other symptoms accompanying your sleeplessness? | Did the pharmacist ask about any actions you have already taken (if any)? | 0.176 | 0.005 | Weak positive correlation |
|  | Did the pharmacist ask about the presence of any chronic health problems? | 0.296 | 0 | Weak positive correlation |
|  | Did the pharmacist introduce himself/herself? | 0.244 | 0 | Weak positive correlation |
|  | Was your privacy ensured during the consultation? | 0.251 | 0 | Weak positive correlation |
|  | Did the pharmacist explain the reason for asking questions? | 0.236 | 0 | Weak positive correlation |
|  | Did the pharmacist ask if you needed any additional information or had any questions? | 0.439 | 0 | Weak positive correlation |
|  | Did the pharmacist consider your preferences? | 0.149 | 0.018 | Weak positive correlation |
|  | Did the pharmacist check your understanding of the recommendations? | 0.34 | 0 | Weak positive correlation |
|  | Did the pharmacist offer a way to follow up? | 0.29 | 0 | Weak positive correlation |
|  | Did the pharmacist tell you when you should visit a doctor? | 0.237 | 0 | Weak positive correlation |
| Did the pharmacist ask about any actions you have already taken (if any)? | Did the pharmacist ask about the presence of any chronic health problems? | 0.206 | 0.001 | Weak positive correlation |
|  | If a product was provided, provide the name and the strength of the product. | -0.156 | 0.013 | Weak negative correlation |
|  | Did the pharmacist introduce himself/herself? | 0.253 | 0 | Weak positive correlation |
|  | Was your privacy ensured during the consultation? | 0.16 | 0.011 | Weak positive correlation |
|  | Did the pharmacist explain the reason for asking questions? | 0.167 | 0.008 | Weak positive correlation |
|  | Did the pharmacist ask if you needed any additional information or had any questions? | 0.17 | 0.007 | Weak positive correlation |
|  | Did the pharmacist check your understanding of the recommendations? | 0.277 | 0 | Weak positive correlation |
| Did the pharmacist ask about the presence of any chronic health problems? | Did the pharmacist introduce himself/herself? | 0.202 | 0.001 | Weak positive correlation |
|  | Was your privacy ensured during the consultation? | 0.144 | 0.022 | Weak positive correlation |
|  | Did the pharmacist explain the reason for asking questions? | 0.17 | 0.007 | Weak positive correlation |
|  | Did the pharmacist ask if you needed any additional information or had any questions? | 0.28 | 0 | Weak positive correlation |
|  | Did the pharmacist consider your preferences? | 0.162 | 0.01 | Weak positive correlation |
|  | Did the pharmacist check your understanding of the recommendations? | 0.275 | 0 | Weak positive correlation |
|  | Did the pharmacist offer a way to follow up? | 0.183 | 0.004 | Weak positive correlation |
|  | Did the pharmacist tell you when you should visit a doctor? | 0.141 | 0.025 | Weak positive correlation |
| Was your privacy ensured during the consultation? | Dispensary Load (Number of customers at the time of visit) | 0.397 | 0 | Weak positive correlation |
| Did the pharmacist introduce himself/herself? | Did the pharmacist avoid using inappropriate language? | -0.127 | 0.045 | Weak negative correlation |
| Did the pharmacist have a professional appearance? | Did the pharmacist avoid using inappropriate language? | 0.19 | 0.003 | Weak positive correlation |
| Did the pharmacist avoid using inappropriate language? | Did the pharmacist ask if you needed any additional information or had any questions? | -0.127 | 0.045 | Weak negative correlation |
| Did the pharmacist check your understanding of the recommendations? | If a product was provided, provide the name and the strength of the product. | -0.13 | 0.04 | Weak negative correlation |
| Did the pharmacist offer a way to follow up? | If a product was provided, provide the name and the strength of the product. | -0.183 | 0.004 | Weak negative correlation |
| Did the pharmacist tell you when you should visit a doctor? | Did the pharmacist introduce himself/herself? | 0.128 | 0.043 | Weak positive correlation |
